# Neurodegenerative Disease: From Molecular Basis to Therapy, 3rd Edition

**DOI:** 10.3390/ijms27114926

**Published:** 2026-05-29

**Authors:** Claudia Ricci

**Affiliations:** Department of Medical, Surgical and Neurological Sciences, University of Siena, 53100 Siena, Italy; claudia.ricci@unisi.it

Neurodegenerative diseases encompass a wide range of disorders linked to aging, predominantly impacting the central nervous system and leading to progressive neural deterioration. As the population continues to age, neurodegenerative diseases are becoming more prevalent, placing substantial financial pressure on healthcare systems. The majority of currently available treatments focus on managing symptoms rather than addressing the root causes, having little to no meaningful effect on slowing their progression.

The advancements in neurobiology and neurogenetics have enhanced our understanding of the pathogenesis of neurodegenerative diseases. These breakthroughs have enabled the development of molecularly targeted therapies designed to halt or slow the core pathological processes that cause neuronal damage and, consequently, cognitive and motor impairments. In some cases, these conditions are associated with genetic variants or disruptions in cellular pathways. Researchers have identified shared mechanisms among various neurodegenerative disorders, including the formation of misfolded protein aggregates, abnormal protein accumulation, neuroinflammation, and genetic and epigenetic influences. However, a precision medicine approach is becoming increasingly necessary to capture the variability between individuals and disease subtypes. A deeper understanding of these mechanisms is crucial for developing effective future treatments. Despite notable advancements in our understanding of the key processes involved in the development of these diseases, applying this knowledge to clinical practice remains in its early stages.

This Special Issue aims to provide a comprehensive update on the recent advancements in neurodegenerative disease research, from understanding their molecular basis to developing new therapies. This Special Issue includes a range of papers exploring the molecular mechanisms and pathways driving neurodegenerative conditions, alongside studies highlighting potential therapeutic targets and introducing novel drug candidates for specific diseases. Additionally, this Special Issue includes reviews focusing on the pathogenic processes and biomarkers associated with common neurodegenerative disorders, such as Alzheimer’s disease and Parkinson’s disease (PD).

Several papers describe new potential therapeutic treatments. Shimozono et al. [1] explored novel therapeutic approaches to spastic paraplegia 80 (SPG80), a pure form of juvenile-onset hereditary spastic paraplegia (HSP) caused by mutations in the ubiquitin-associated protein 1 (*UBAP1*) gene. They assessed the therapeutic potential of 4-phenylbutyric acid (4-PBA), a chemical chaperone and histone deacetylase inhibitor, as a treatment in a mouse model expressing a disease-associated truncated UBAP1 variant (Ubap1 knock-in (KI) mice). Administering 4-PBA significantly increased the motor performance of KI mice in the rotarod and beam walk tests. The maximal benefits were obtained when treatment was started in the pre- or early-symptomatic stages; however, partial efficacy was also observed when treatment began after symptom onset. In addition, 4-PBA reduced the activation of spinal microglia and partially restored their morphology, although astrocytic reactivity did not change. These findings support 4-PBA as a treatment for SPG80 and show the potential of future therapeutic strategies that target proteostasis in HSP.

Ansari et al. investigated a potential treatment for Alzheimer’s disease, focusing on amnesia, which involves the loss of short-term memory and the inability to retain facts, information, and experiences [2]. Amnesia is a primary symptom of dementia [3]. They explored the therapeutic potential of sobrerol (coded as NRM-331) in a scopolamine-induced amnesia mouse model, paying attention to its ability to improve memory deficits and enhance neuronal plasticity. Sobrerol is a synthetic mucolytic agent derived from terpenes with anti-inflammatory and antioxidant properties that has recently emerged as a potential treatment for multiple sclerosis [4]. Sobrerol may have additional, previously unreported pharmacological effects. The authors conducted multiple behavioral assessments, including the Y-maze, the passive avoidance, and the Morris water maze tests. They also biochemically analyzed serum samples (Aβ 1-40 and Aβ 1-42) and brain tissue (ACh and AChE), as well as histopathologically examined the brain using Nissl staining and tau-IHC. NRM-331 demonstrated an antiamnesic effect, likely due to its capacity to enhance cholinergic signaling in the hippocampus, combined with its antitau and anti-Aβ-synthesis actions. These findings indicate that NRM-331 shows potential as a therapeutic option for individuals with neurodegenerative conditions such as Alzheimer’s disease. By targeting critical factors associated with memory loss and neuronal damage, NRM-331 could open avenues for developing multifunctional treatments incorporating NRM-331 to address Alzheimer’s disease and other related cognitive disorders.

Liu et al. [5] describe a novel small molecule that enhances stable dopamine delivery to the brain in models of PD. PD is a progressive neurodegenerative disorder characterized by motor and nonmotor symptoms. PD is associated with the loss of dopamine in the neurons of the nigrostriatal pathway and the accumulation of misfolded alpha-synuclein. Although levodopa is the gold standard for managing PD symptoms, levodopa does not address the underlying progression caused by alpha-synuclein aggregation, pathological oxidative stress, brain inflammation, or the loss of dopamine neurons. Furthermore, motor fluctuations persist as a major challenge for patients, often arising from variability in levodopa absorption, plasma concentrations, and brain penetration. To address this critical medical issue, Liu et al. focused on a newly synthesized compound, named Pegasus [6], which demonstrated potential in addressing the multifactorial causes of PD. Pegasus is a small molecule with a molecular weight of 652 Da, formed by linking dopamine to a nonantibiotic derivative of doxycycline. Notably, the calculated physicochemical properties suggest that Pegasus is unlikely to cross the blood–brain barrier (BBB) via passive diffusion [7]. The authors obtained novel findings on the mechanisms through which dopamine is delivered to the brain. Following peripheral administration, Pegasus undergoes in vivo metabolism, breaking down into dopamine and tetracycline/doxycycline derivatives, which substantially enhance dopamine delivery. In mice harboring the human A53T mutation of alpha-synuclein, treatment with 1-methyl-4-phenyl-1,2,3,6-tetrahydropyridine (MPTP) caused motor symptoms resembling PD, whereas Pegasus was able to restore motor function and reduce anxiety-like behavior. Furthermore, Pegasus treatment reduced the levels of soluble and insoluble alpha-synuclein, protected dopamine-producing neurons, and reduced astrocytic activation in A53T mice. In vivo toxicity, pharmacology and toxicokinetic studies are still in the preliminary stages; however, these findings collectively suggest that Pegasus is a promising treatment for addressing the challenges associated with levodopa dosing.

Another key area of neurodegeneration research is identifying new drug targets. Ferrari et al. conducted a whole-transcriptome analysis of post mortem brain tissue samples from individuals with Alzheimer’s disease, exploring the molecular mechanisms that drive the region-specific distribution of Alzheimer’s disease pathology at various stages of progression [8]. Samples were obtained from the hippocampus (HI), temporal cortex (TC), parietal cortex (PC), cingulate gyrus (CG) and substantia nigra (SN). The samples were then compared those from individuals without dementia. The transcriptomic results revealed a higher abundance of differentially expressed genes (DEGs) in the TC and CG, and a lower abundance of DEGs in the HI, PC and SN. Notably, the shared DEGs primarily involved in synaptic transmission were significantly downregulated in the HI and TC, whereas genes primarily involved in protein folding and trafficking were significantly downregulated in the PC, CG, and SN. These findings imply that the AD progression follows a specific pattern related to time and severity, beginning with protein misfolding in the PC, CG, and SN and resulting in synaptic impairment in the HI and TC. The study suggests that a map outlining the molecular and biological processes involved in AD pathogenesis can be constructed. This map could facilitate the identification of novel biological targets for the development of effective and timely therapeutic approaches.

The patient must be characterized to identify the most suitable therapeutic strategy. Nowakowska et al. applied this approach to evaluate the risk of postoperative delirium in patients who had undergone cardiac surgery [9]. Delirium is commonly observed after major surgery, especially in older patients with physical and psychiatric illnesses or impaired cognition [10]. Postoperative delirium is associated with a poor prognosis, an increased risk of death, and impaired functioning, even after the syndrome has resolved [11]. Although the risk factors for delirium are well-understood, research into the pathophysiology of delirium has only recently begun. Authors investigated whether specific microRNAs (miRNAs) are associated with an increased risk of delirium They selected miRNAs related to brain function and development: miR-9-3p, miR-34c-5p, miR-96-5p, miR-183-5p and miR-374-3p. Serum samples that were obtained one day before and the day after surgery from 60 patients with delirium and 60 randomly selected individuals without delirium were analyzed. Univariate comparisons revealed that the preoperative levels of miR-96-5p and miR-183-5p, as well as postoperative levels of miR-34c-5p, miR-96-5p and miR-183-5p, were associated with an increased risk of postsurgery delirium. However, multivariate logistic regression showed that only the miR-183-5p level was an independent predictor of delirium. Decreased expression levels of miR-183-5p independently predisposed patients who underwent cardiac surgery to postoperative delirium. This association may be linked to increased oxidative stress and neuroinflammation in this patient group. This finding could inform subsequent actions based on a precision medicine strategy.

Research into neurodegeneration remains crucial, as a further understanding of the condition could lead to the development of new treatments and more effective approaches. Therefore, the majority of papers in this Special Issue logically address various aspects of the mechanisms underlying neurodegeneration. Three studies focused on this topic. Razak et al. studied diabetic peripheral neuropathy (DPN), which is one of the most prevalent and severe complications of diabetes [12]. DPN affects a large proportion of individuals with diabetes and can result in a range of debilitating outcomes, from early symptoms such as paresthesia to more severe complications such as limb loss and death [13]. DPN is characterized by Schwann cell dysfunction, demyelination, and impaired nerve regeneration. To deepen our understanding of the mechanisms responsible for Schwann cells modifications under hyperglycemic conditions, the authors studied the effects of high glucose treatment on the phenotype of Schwann cells, as well as assessing the involvement of the mTOR signaling pathway. The authors found that high-glucose conditions (50 mM) considerably reduce the expression of myelin basic protein (MBP), accompanied by the significant upregulation of the expression of total mammalian target of rapamycin (mTOR) and a concurrent reduction in the phosphorylation of mTOR at Ser2448. This finding suggests that hyperglycemic stress initiates Schwann cell dedifferentiation and dysregulates mTOR signaling. In addition, increased c-Jun and p75NTR expression levels, along with decreased Krox-20 levels, suggests a shift toward a repair-like phenotype, a process closely associated with demyelination and impaired nerve support. Taken together, these results show that impaired myelin protein expression, phenotypic changes associated with repair, and the altered activity of the mTOR pathway are key features of Schwann cell responses to hyperglycemia and contribute to the development of neuropathy.

Sánchez et al. explored the initial factors that contribute to Niemann–Pick disease type C (NPC), a genetic neurodegenerative disorder caused by mutations in the *NPC1* and/or *NPC2* genes [14]. This condition is marked by the selective vulnerability of specific cells, with cerebellar anterior Purkinje neurons being particularly affected. These neurons exhibit a specific degenerative pattern associated with the loss of NPC1 and/or NPC2 protein function, gradually progressing into the posterior regions of the cerebellum. Through the bioinformatics analysis of presymptomatic Npc1−/−Purkinje neurons in mice and in vitro testing on primary dermal fibroblasts from NPC patients, the researchers found that Purkinje neurons in the anterior cerebellum are particularly susceptible at an early stage due to oxidative stress and disruptions in iron homeostasis. The vulnerability to oxytosis/ferroptosis was higher in lobules I–III than in lobules VI and X, consistent with the sublethal threshold model of neurodegeneration in NPC. NPC-affected cells exhibited diminished antioxidant defenses, such as reduced levels of the enzyme GPx4, rendering the cells incapable of counteracting lipid peroxidation. The study suggests that antiferroptotic compounds can be used as innovative therapeutics to slow neurodegeneration, providing a new approach to treating NPC and other neurodegenerative disorders associated with oxytosis/ferroptosis.

The aggregation and accumulation of α-synuclein (α-Syn) are well-established contributors to the pathogenesis of PD. However, the involvement of the BBB in the mechanisms for clearing α-Syn remains poorly understood. Yokoya et al. focused on elucidating this involvement by examining the functional role of pericytes, a key cellular component of the BBB with the capacity to degrade various forms of α-Syn [15]. Utilizing primary cultures derived from rat pericytes, brain endothelial cells, and astrocytes, the authors found that, following α-Syn uptake by these cell types, the levels of intracellular α-Syn significantly decreased exclusively within pericytes. This pericyte-specific decrease in α-Syn was inhibited by treatment with the autophagy inhibitor bafilomycin A1 and the proteasome inhibitor MG132. Knocking down degradation enzymes or familial PD-associated genes such as cathepsin D, DJ-1 and LRRK2 did not affect α-Syn clearance. However, treatment with pharmacological inhibitors Akt, ERK and p38 MAPK inhibited the α-Syn degradation by pericytes. These findings suggest that α-Syn degradation by pericytes is mediated by the autophagy–lysosome and ubiquitin–proteasome systems via the α-Syn-activated Akt, ERK and p38 MAPK signaling pathways. From this perspective, these mechanisms represent potential therapeutic targets for developing disease-modifying strategies to halt the progression of α-Syn pathology in PD and other synucleinopathies.

Several reviews also offer in-depth analyses of the specific molecular mechanisms associated with neurodegeneration. Toader et al. conducted a comprehensive review describing the current state of research examining the molecular mechanisms through which neurodegeneration occurs and developing therapeutic strategies [16]. The authors examined the intricate network of molecular disruptions that underlie diseases such as Alzheimer’s disease, PD, amyotrophic lateral sclerosis (ALS), and Huntington’s (HD). Despite their unique clinical manifestations, these diseases share pathological features including protein misfolding and aggregation, neuroinflammation, the prolonged activation of brain immune cells, mitochondrial dysfunction and oxidative stress, as well as genetic and epigenetic factors. These mechanisms often act in intertwined pathways that drive the progression of these diseases. The review summarizes the most promising advances in identifying molecular biomarkers and explores innovative therapies emerging from cutting-edge research. Among these therapies are nanotechnology-driven drug delivery systems capable of crossing the BBB, CRISPR gene-editing tools developed to correct harmful genetic mutations, and stem cell techniques aimed at replacing damaged neurons and fostering neuroprotective conditions. In this review, attention is paid to precision medicine, where pharmacogenomics is transforming personalized treatment, enabling therapies tailored to individual genetic profiles. Molecular diagnostics and biomarkers are ushering in an era of early, precise disease detection. Additionally, emerging insights into the gut–brain axis are gaining attention, as evidence increasingly indicates that microbiome modulation could help mitigate the neuroinflammatory responses associated with the advancement of neurodegenerative conditions. In summary, this review emphasizes how molecular research is prompting a paradigm shift in the treatment of neurodegenerative diseases by providing novel therapeutic targets and facilitating more precise, multitargeted approaches. By addressing the web of interconnected mechanisms underlying these diseases, progress is being achieved toward treatments that exceed symptom management to intervene at critical stages of disease progression. This research is paving the way for ensuring neurodegenerative conditions are more manageable as well as potentially preventable in the future.

Ruiz-Sanchez et al. focused on a key component in neurodegeneration: nuclear receptor subfamily 4 group A member 2 (NR4A2) [17]. This transcription factor regulates the expression of genes involved in biological processes, such as cell proliferation, neuronal development, the immune response, cellular stress, apoptosis, DNA repair, and angiogenesis. *NR4A2* belongs to the class of genes known as immediate early genes, which are rapidly and transiently activated within minutes to an hour in response to cellular stimuli. Reduced *NR4A2* gene expression levels are associated with neurodegenerative and psychiatric disorders, including PD, Alzheimer’s disease progression, schizophrenia, substance abuse (alcohol and amphetamines), neurodevelopmental disorders, and cognitive impairment. This review provides a comprehensive summary of the current understanding of the *NR4A2* gene, focusing on its structure and the molecular processes involved in regulating its expression. Special attention is paid to the primary epigenetic mechanisms influencing *NR4A2* expression, such as DNA methylation, histone deacetylation, and microRNA regulation. Additionally, the discussion highlights the gene’s involvement in central nervous system disorders linked to disruptions in its expression. The authors conclude by exploring the potential of these regulatory mechanisms as biomarkers and therapeutic targets for addressing neurodegenerative diseases and psychiatric disorders.

Epigenetics is a cutting-edge field of neurodegenerative disease research. Among the various epigenetic mechanisms, histone acetylation has been identified as an epigenetic modification that directly affects gene transcription. Zhang et al. reviewed the role of histone lactylation in neurological disorders such as Alzheimer’s disease, depression, neuroinflammation, and aging [18]. This review closes a gap, as previous reviews have summarized histone lactylation in cancer and immunology. The authors accurately describe the specific mechanisms and sites of histone lactylation, including lactylation and delactylation. The authors further examine the involvement of histone lactylation in a range of neurological disorders. In Alzheimer’s disease, for example, histone lactylation activates the transcription of glycolysis genes, creating a positive feedback loop that intensifies the activation and dysfunction of microglial cells, thereby aggravating the pathological condition. This loop involves active glycolysis, H4K12la, and pyruvate kinase M2 (PKM2). However, inhibiting PKM2 disrupts the positive feedback loop involving glycolysis, H4K12la, and PKM2 in microglia. This reduces microglial activation and neuroinflammatory responses [19,20]. Moreover, astrocyte–neuron lactate transport modulates synaptic plasticity via BDNF lactylation, a process required for the formation of long-term memory [21]. Depression is characterized by the release of lactate by cortical astrocytes under fluoxetine/paroxetine treatment and by a reduction in astrocyte density in the prefrontal cortex due to chronic stress. These characteristics suggest that the dysregulation of astrocytic lactate is a core component of the etiology of depression [22,23]. These findings could affect future therapeutic approaches. However, the authors underline that the understanding of the role and regulation of histone lactylation in the brain is still developing, and further research is needed to completely understanding the therapeutic potential of this mechanism.

In their review, Elazar et al. focus on the importance of serine metabolism in the brain, specifically referencing the role of solute carrier family 1 member 4 (SLC1A4) in maintaining serine homeostasis during neurodevelopment and brain aging [24]. SLC1A4 is a neutral amino acid transporter, also known as alanine-serine-cysteine transporter 1 (ASCT1), which is encoded by the *SLC1A4* gene. In the brain, SLC1A4 transports the L- and D-isomers of serine. L-serine is involved in many cellular processes, including protein and sphingolipid synthesis. D-serine acts as a co-agonist essential for normal neurotransmission through N-methyl-D-aspartate receptors, which are fundamental for synaptic plasticity, learning, and memory. SLC1A4 plays a pivotal role in promoting and preserving brain health throughout life by facilitating the transport of L-serine across the BBB and regulating synaptic D-serine levels. The authors explore the role of SLC1A4 in neurodevelopment and neurodegeneration, evaluating the potential therapeutic benefits of serine supplementation in treating various conditions, including symptoms associated with SLC1A4 mutations in spastic tetraplegia, thin corpus callosum, and progressive microcephaly (SPATCCM), as well as neuropsychiatric disorders such as schizophrenia and depression, traumatic brain injury, and neurodegenerative diseases such as Alzheimer’s disease and PD. The authors emphasize that, given the role of the SLC1A4 transporter in mediating the exchange of intracellular L-serine for extracellular D-serine, SLC1A4 may represent a key therapeutic target for maintaining serine homeostasis in the brain. Furthermore, when D-serine levels are reduced, the activity of SLC1A4 could be reduced using selective, high-affinity inhibitors that modulate the transporter’s function. Moreover, identifying the pathways that regulate SLC1A4 levels and activity may reveal methods of increasing these levels to treat diseases associated with elevated levels of extrasynaptic D-serine. However, further studies are needed to assess potential side effects.

Finally, Athanasaki et al. [25] reviewed the molecular biomarkers used to diagnose Alzheimer’s disease and determine eligibility for new disease-modifying treatments, such as the two antiamyloid antibodies currently approved by the FDA and EMA, lecanemab [26] and donanemab [27]. Rather than providing a comprehensive review, the authors highlight the ongoing discrepancies among the various existing guidelines. The authors consider various types of biomarkers—including amyloid (A) and tau (T) biomarkers in cerebrospinal fluid and plasma, biomarkers of neurodegeneration and neuroinflammation, and genetic biomarkers such as APOE—and note that hybrid ratios such as τP-181/Aβ42 and plasma τP-217/Aβ42 enhance diagnostic discrimination. A key point of contention is whether amyloid positivity (A+) alone is sufficient for diagnosing AD and accessing treatments or whether tau positivity (T+) is also required. The authors conclude that no satisfactory consensus currently exists, as conflicting diagnoses can arise in approximately 42% of patients when only one biomarker is abnormal. A diagnosis based solely on biomarkers is insufficient: comorbidities, clinical phenotype, and the predominantly European origin of the analyzed data—which limit generalizability—remain essential considerations. Overcoming these limitations will be key to more accurately identifying eligible patients and advancing a personalized approach to care.

## Conclusions

The papers collected in this Special Issue reflect the breadth and dynamism of the current neurodegenerative disease research. Together, these studies illustrate the progression in multiple interconnected domains. These domains include the molecular analysis of pathogenic mechanisms and the identification of novel therapeutic targets, the development of promising drug candidates, and the refinement of diagnostic biomarker frameworks.

A recurrent theme is the complexity and heterogeneity of neurodegeneration. The evidence indicates the involvement of multiple pathways rather than a single cascade. This complexity underscores the necessity for targeted therapeutic strategies and a precision medicine approach that accounts for the variability among individuals and disease subtypes. Several papers highlight the translational potential of new molecular insights. Research using animal models and post mortem tissue is beginning to produce pharmacological candidates, including 4-PBA in hereditary spastic paraplegia, Pegasus in PD, and antiferroptotic compounds in Niemann–Pick disease. Additional research could result in these compounds being adopted in clinical settings. The increasing complexity of biomarker research, including the ongoing discussion on amyloid and tau profiles in Alzheimer’s disease, shows the importance of correctly identifying patients before any treatment can be properly assessed. Additionally, novel regulatory mechanisms, such as histone lactylation, NR4A2 transcriptional control, and SLC1A4-mediated serine transport, have emerged. These provide insight into disease biology and could be exploited as therapeutic targets. These findings suggest that more opportunities exist for intervention than the classical pathological hallmarks suggest. Nevertheless, several challenges remain to be addressed. The process of translating laboratory findings into effective clinical treatments remains slow, and many of the biomarkers and targets discussed in this Special Issue require further validation, particularly in relation to diverse patient populations.

In conclusion, this Special Issue provides a timely and comprehensive overview of the current state of neurodegeneration research. The insights presented here will hopefully contribute to a deeper understanding of these devastating diseases and inspire future studies that bring us closer to treatments capable of not only alleviating symptoms but also fundamentally altering the course of neurodegeneration.
